# Development of a patient reported outcome scale for fatigue in multiple sclerosis: The Neurological Fatigue Index (NFI-MS)

**DOI:** 10.1186/1477-7525-8-22

**Published:** 2010-02-12

**Authors:** Roger J Mills, Carolyn A Young, Julie F Pallant, Alan Tennant

**Affiliations:** 1 The Walton Centre for Neurology and Neurosurgery, Liverpool, L9 7LJ, UK; 2 School of Rural Health, University of Melbourne, 49 Graham St, Shepparton, Victoria, 3630, Australia; 3 Department of Rehabilitation Medicine, Faculty of Medicine and Health, University of Leeds, D Floor, Martin Wing, Leeds General Infirmary, Gt George Street, Leeds, LS1 3EX, UK

## Abstract

**Background:**

Fatigue is a common and debilitating symptom in multiple sclerosis (MS). Best-practice guidelines suggest that health services should repeatedly assess fatigue in persons with MS. Several fatigue scales are available but concern has been expressed about their validity. The objective of this study was to examine the reliability and validity of a new scale for MS fatigue, the Neurological Fatigue Index (NFI-MS).

**Methods:**

Qualitative analysis of 40 MS patient interviews had previously contributed to a coherent definition of fatigue, and a potential 52 item set representing the salient themes. A draft questionnaire was mailed out to 1223 people with MS, and the resulting data subjected to both factor and Rasch analysis.

**Results:**

Data from 635 (51.9% response) respondents were split randomly into an 'evaluation' and 'validation' sample. Exploratory factor analysis identified four potential subscales: 'physical', 'cognitive', 'relief by diurnal sleep or rest' and 'abnormal nocturnal sleep and sleepiness'. Rasch analysis led to further item reduction and the generation of a Summary scale comprising items from the Physical and Cognitive subscales. The scales were shown to fit Rasch model expectations, across both the evaluation and validation samples.

**Conclusion:**

A simple 10-item Summary scale, together with scales measuring the physical and cognitive components of fatigue, were validated for MS fatigue.

## Background

One of the symptoms causing the greatest morbidity and disability in multiple sclerosis (MS) is fatigue [1,2]. It has been suggested that health services should apply a broad range of approaches and repeatedly assess fatigue in persons with MS, to provide preventive care and appropriate interventions [3]. However, assessing fatigue is not easy since the symptom is inherently complex and the pathophysiology is not well explained [4,5]. A major problem has been the absence of a clear definition of fatigue [5-7] and, consequently, there is debate regarding the possible dimensionality of the phenomenon, with some arguing that fatigue can only be understood as a multidimensional entity,[8] while others argue that it is unidimensional [9]. This immediately poses a problem for quantification of fatigue, since an unambiguous definition and unidimensionality are fundamental requirements of measurement.

Regardless of these issues, several scales to measure fatigue have been developed. For example, the Fatigue Severity Scale (FSS)[4] has been one of the most widely used fatigue scales for MS and, true to its origins, has often been employed to dichotomise groups into those with 'normal' levels of fatigue and those where fatigue had a disproportionately high impact. Another scale, the Modified Fatigue Impact Scale (MFIS)[10] has been recommended by the MS Council as an outcome measure for fatigue [5]. Despite their widespread use, some limitations have recently been observed with respect to these scales, suggesting that they do not satisfy modern standards of outcome measurement [11,12]. Such deficiencies suggest a need for a better definition of, and a high-quality measurement instrument for, fatigue [6]. Fatigue has been defined, as a result of qualitative analysis, as a:

'reversible motor and cognitive impairment with reduced motivation, and a desire to rest, either appearing spontaneously or brought on separately by mental or physical activity, humidity, acute infection and food ingestion. It was relieved by daytime sleep or rest without sleep. It could occur at any time but was usually worse in the afternoon'[6].

In MS, fatigue could be daily, had usually been present for years and had greater severity than any pre-morbid fatigue. It was a synthesis of the features, which arose from that qualitative analysis, which defined the symptom and full details of this can be found elsewhere [6].

### Objective

The current study takes this qualitative work forward to the next phase of measurement, with the aim of developing a valid and reliable patient reported outcome scale for fatigue, the Neurological Fatigue Index (NFI-MS).

The items in the scale are based on the previous qualitative work. Table 1 provides some example of how the items relate to the thematic framework of the definition. The scale was developed to conform to Rasch measurement model standards,[13] and the U.S. Food and Drug Agency's (FDA) guidelines for the development of patient-reported outcome measures [14].

**Table 1:** Item origins.

Framework	Feature	Item wording
SE motor features	can develop weakness	Sometimes, I lose my body strength
SE cognitive features	concentrate on simple tasks	Sometimes, I really have to concentrate on what are usually simple things
SE motivation	thought puts off doing	The thought of having to do something often puts me off doing it
SE tiredness	tiredness	By the end of the day I'm shattered
Cadence	carry over	If I've overdone things, I know about it the next day
Precipitating/aggravating factors	physical exertion induces weakness	I soon become weak after physical effort
Relieving factors	day rest restorative	Resting allows me to carry on
Severity	weak at rest	I can become weak even if I've not been doing anything
Associated features	unrefreshing nocturnal sleep	When I awake in the morning, I feel unrefreshed

Examples of item wording representing the individual features of fatigue in the context of the thematic framework derived from the qualitative analysis. SE = subjective experience.

## Methods

The study had approval from relevant local research ethics committees (Sefton EC115.03 and Hammersmith 05/Q0401/7). All subjects received written information on the study and gave written informed consent prior to participation.

### Sample and materials

Initially, there were 57 potential items for the new scale each with a common four point, Likert-style response option [15] of 'strongly disagree', 'disagree', 'agree' and 'strongly agree', with each item being scored 0, 1, 2, 3. There was a single sentence instruction at the start of the scale asking respondents to consider their experience over the previous two weeks. Emphasis was placed on the dynamic quality or reversible nature of fatigue *e.g*., my limbs *can become *heavy rather than my limbs *are *heavy, in order that the scale should not be confounded by fixed neurological deficit. The nascent scale was put to an expert, multidisciplinary panel of ten professionals experienced in MS and fatigue, comprising: MS specialist nurses, MS specialist physiotherapists and occupational therapists, consultants in neurology and neurorehabilitation each with specialist interest in MS, a consultant rheumatologist and a clinical physiologist in sleep medicine, in order to confirm that items and their wording were reasonable.

The draft scale was subsequently administered, face-to-face, to 15 MS patients in the outpatient clinic. They were encouraged to give a running commentary during completion. This allowed identification and remedy of any gross problems with wording or item dysfunction. They were also asked to comment on the completeness of the item pool, and if any obvious features had been omitted.

A random cross-sectional cohort of 1223 patients with clinically definite MS,[16] identified from research databases in two centres in the UK (WCNN, Liverpool and Imperial College Healthcare Trust, London) was then sent packs, by mail, containing the set of potential items for the proposed scale, questions on demographics and basic disease information, together with other scales chosen for comparative analysis. Participants of any age, disease type, and disability level were included (the range of Expanded Disability Status Scale scores [17] (EDSS), was 0-9.0 as rated by neurologists at the time of database enrolment). Participants were also asked to estimate their best walking distance from a choice of four options, in order to corroborate EDSS at the time of questionnaire completion.

The additional scales in the questionnaire pack were:

i) Visual analogue scale (VAS): a 10 cm, modified (*i.e*. marked with cm gradations), horizontal visual analogue scale with anchors of 'lively and alert' (zero, left) and 'absolutely no energy to do anything at all' (10, right).

ii) Fatigue Severity Scale-5 (FSS-5): a short-form of the original nine item scale, including five items with a seven-point response option, modified from the original Rasch analysis in an MS population [11] iii) Modified Fatigue Impact Scale, Phys-8 and Cog-5: an eight item physical scale and a five item cognitive fatigue scale modified from the original MFIS subscales by Rasch analysis in an MS population [12].

Retesting was performed at 2 to 4 weeks.

### Psychometric analysis/item reduction

#### Initial exploration of dimensionality

Given the multi-faceted nature of fatigue that had previously emerged from the qualitative analysis, and consistent with some of the published literature about the dimensionality of fatigue,[8] an exploratory factor analysis was undertaken to identify potential domains of fatigue. A Principal Components Analysis (PCA), based on a polychoric correlation matrix, was undertaken to extract the factors followed by oblique rotation of factors using Oblimin rotation (delta = 0). Suitability of the data for factor analysis was tested by Bartlett's Test of Sphericity,[18] which should be significant, and the Kaiser-Meyer-Olkin (KMO) measure of sampling adequacy, which should be >0.6[19,20]. The number of factors to be retained was guided by three decision rules: Kaiser's criterion (eigenvalues above 1);[21] inspection of the screeplot,[22] and by the use of Horn's parallel analysis [23]. Parallel analysis is one of the most accurate approaches to estimating the number of components [24]. The size of eigenvalues obtained from PCA are compared with those obtained from a randomly generated data set of the same size. Only factors with eigenvalues exceeding the values obtained from the corresponding random data set are retained for further investigation. Parallel analysis was conducted using the software developed by Watkins [25].

Items identified to be associated in domains were taken forward to the Rasch analysis, to be analysed on a domain-specific basis and also to test if an overall summary scale could be derived.

#### Rasch Analysis

Rasch analysis is a modern psychometric approach which is widely used in the development, refinement and evaluation of patient reported outcome measures [13,26-28]. The Rasch model states that the probability of a person giving a certain answer to an item is a logistic function of the difference between the person's ability (in this case level of fatigue) and the item's difficulty (in this case the level of fatigue expressed by the item)[13]. Where the observed pattern of responses do not deviate too much from that expected by the model, the scale is said to satisfy Rasch model expectations. Full details of the process of Rasch analysis are given elsewhere [29,30]. Briefly, the process is concerned with whether or not the data meet the model expectations, and provides an assessment of the suitability of the response scale, the fit of individual items, item bias, and the dimensionality and targeting of the scale as a whole.

In summary, fit of data to the Rasch model was deemed acceptable if the following criteria were fulfilled:

1) ordered item category thresholds;

2) assumption of local independence holds (no significant (>0.3) correlations in the residuals), reflecting that once account of the trait under consideration has been taken, the items do not display any further associations that would indicate redundancy or multidimensionality;

3) assumption of probabilistic ordering of items holds, determined by a range of fit statistics:

a. both total chi-square probability and individual item chi-square probability values non-significant (5% alpha with Bonferroni correction for the number of items);

b. individual item fit residual, by convention, within ± 2.5 (99% CI);

c. mean and SD of both summary item fit residual and person fit residuals approaching 0 and 1 respectively;

4) reliability (person-item separation index) greater than 0.85;

5) differential item functioning (DIF) absent for age, sex and disease duration as defined by a non-significant ANOVA (5% alpha with Bonferroni correction). Where necessary, DIF was tested to see if it cancelled out at the test level [31]. In addition, DIF was used to test invariance of measurement across time in the test-retest analysis;

6) Strict unidimensionality assessed by comparing person estimates from two sets of items derived from the positive and negative loadings of the first component in PCA of the residuals. Unidimensionality is indicated if less than 5% of t-tests are significant (or the lower bound of the binomial confidence interval overlaps 5%)[32,33].

The unrestricted (partial credit) Rasch polytomous model was used with a conditional pair-wise parameter estimation [34]. Failure of items to fit Rasch model expectations led to an iterative procedure using techniques for collapsing response categories, item deletion, and adjusting for DIF where necessary.

For Rasch analysis, a sample size of 243 will provide accurate estimates of item and person locations irrespective of the scale targeting [35]. Assuming a 50% response rate from the mail-out, that sample size would allow the data to be split randomly into two equal samples, one for the initial evaluation of the data set, the second to validate the results.

#### External comparison

Linear correlation of the Rasch derived interval level person estimates, from the new scale, was performed with the comparator measures, having also been transformed to interval scaling by Rasch analysis. Consequently, Pearson correlation coefficients were used between these estimates except for the VAS, which remained as an ordinal scale, and so Spearman correlation was used. All correlations were expected to be moderate (0.4-0.7) in size.

#### Test-Retest Reliability

The test-retest reliability of scales was undertaken with Spearman correlation on un-transformed data (to reflect how it is most likely to be used in a clinic setting). Values of ≥ 0.7 are considered appropriate. In addition, median values are reported at both time points and their differences tested by a Wilcoxon Signed Rank test.

#### Raw-Score to Interval scale conversion

Given fit to the Rasch model, a straightforward conversion is available between the raw score for each scale, and the interval scale estimate provided by the model (the person location), in logits. The logit estimates are converted to the same range as the raw score by a further simple linear transformation. This nomogram can be used to obtain linear estimates from the raw scores of other samples only when their data are complete.

The Rasch analysis was performed using the RUMM 2020 software [36]. All other analysis was undertaken with SPSS version 15.

## Results

### Review panel and cognitive debriefing

All items were confirmed as being reasonable by the review panel; one additional item regarding morning sleep inertia was added. During the cognitive debriefing, six items were discarded because it was clear that they would not be relevant to all patients (*e.g*. reference to relapse and long journeys) and two items were reworded, producing a 52 item scale. Table 1 illustrates some of the pool items in the context of both the individual features of fatigue and the wider framework of the qualitative analysis.

### Sample characteristics

635 packs were returned (635/1223, 51.9% response). 451 (71%) were female. Mean age was 46.6 years (SD 10.9, range 21-83), 54 (8.5%) had primary progressive disease, 337 (53.1%) relapsing remitting and 177 (27.9%) secondary progressive disease, 67 (10.6%) had unknown disease type. The mean duration of MS was 15.1 years (SD 9.5, range 2-49). There was a wide range of EDSS scores (0-9.0).

### Psychometric analyses

The main sample was split randomly into two, making an 'evaluation' and a 'validation' sample. Comparison of these samples by t-test or chi-square test across a range of characteristics revealed no significant differences (Table 2). A further 151 subjects completed the retest at 2-4 weeks.

**Table 2:** Comparison of the evaluation and validation sample characteristics.

	Characteristic	Evaluation sample	Validation sample	Difference between evaluation and validating sample
	number of subjects	317	318	
	mean age (SD, min.—max.)(yrs)	46.8 (11.3)	46.4 (10.6)	t-test p = 0.606
	number female (%)	234 (73.8)	217 (68.2)	chi-square p = 0.144
	mean disease duration (SD, min.—max.)(yrs)	16.0 (9.7, 2—49)	14.2 (9.4, 2—45)	t-test p = 0.064
disease type, n (%)	pp	25 (7.9)	29 (9.1)	chi-square p = 0.932
	rr	169 (53.3)	168 (52.8)	
	sp	88 (27.8)	89 (28.0)	
	unknown	35 (11.0)	32 (10.1)	
EDSS, n (%)	0—4.0	104 (32.8)	110 (34.6)	chi-square p = 0.88
	4.5—6.5	101 (31.9)	95 (29.9)	
	7.0—7.5	70 (22.1)	66 (20.8)	
	8.0—9.5	38 (12.0)	42 (13.2)	
	unknown	4 (1.3)	5 (1.6)	
	mean 100 mm VAS fatigue score (SD, min.—max.)	55.73 (24.4, 0—100)	52.11 (23.19, 0—100)	t-test p = 0.059

pp = primary progressive, rr = relapsing remitting, sp = secondary progressive, VAS = visual analogue scale.

#### Factor analysis

Bartlett's Test of Sphericity was highly significant (p < 0.001) and the Kaiser-Meyer-Olkin (KMO) measure of sampling adequacy value of 0.94, both supporting the factorability of the matrix. Principal Components Analysis with Oblimin rotation revealed four potential subscales from the 52 item set, which was also supported by parallel analysis. Thirty nine of the 52 items loaded substantially onto these four factors. After removing all items with standardised loadings of less than 0.4, the resulting four factor solution, which explained 62% of the total variance, could be interpreted as representing physical (16 items); relief by diurnal sleep or rest (7 items); abnormal nocturnal sleep and sleepiness (8 items), and cognitive (8 items) (see Table 3).

**Table 3:** Pattern matrix of four factor solution from PCA with Oblimin rotation.

	Component			
**Item**	**1:Physical**	**2: Cognitive**	**3: Diurnal sleep/rest**	**4: Abnormal sleep**

19	.783			
01	.739			
09	.736			
51	.736			
22	.733			
03	.732			
10	.730			
11	.718			
20	.706			
18	.702			
27	.697			
12	.686			
21	.658			
02	.610			
28	.541			-.336
26	.529			
29		-.842		
30		-.828		
17		-.783		
14		-.739		
16		-.716		
13	.389	-.606		
15		-.587		
35		-.452		
39			.780	
40			.769	
42			.705	
43			.632	
41			.625	
07			.549	
05	.397		.538	
44				.757
47				.680
45				.635
46				.573
49				.537
36				.477
06	.305			.417
23				.398

For ease of interpretation only loadings above .3 are displayed.

#### Rasch analysis

Data in the evaluation sample for each of these domains were then fitted to the Rasch measurement model. An iterative process of item reduction involved identifying disordered thresholds, DIF, item misfit and breaches of local dependency, including multi-dimensionality. The summary findings related to the analysis of each domain are given in Table 4.

**Table 4:** Summary fit statistics for Rasch analyses.

Analysis Name	Item Residual		Person Residual		Chi-Square			Uni-dimensional t	% extreme scores in final versions
Evaluation Sample	*Mean*	*SD*	*Mean*	*SD*	*Value*	*p*	PSI	-test (CI)	
1. Physical set up	-0.02	2.353	-0.264	1.385	172	0.056	0.946	14.60% (12.2-17.0)	
2. Physical Final	0.066	0.867	-0.337	1.098	62.8	0.77	0.905	6.03% (3.6-8.4)	8.52%
3. Cognitive Set Up	-0.563	3.058	-0.478	1.362	179.9	<0.001	0.902	6.71% (4.3-9.1)	
4. Cognitive Final	0.21	0.623	-0.432	1.041	24.3	0.665	0.849	4.46%	11.00%
5. Diurnal sleep Set Up	-0.019	0.989	-0.443	1.293	49.7	0.801	0.864	5.75% (3.3-8.2)	
5a. Diurnal sleep modified	-0.069	1.136	-0.451	1.235	68.9	0.083	0.845	4.95%	3.78%
6. Nocturnal Sleep Set Up	0.208	1.873	-0.378	1.379	127.3	<0.001	0.822	5.7 (3.3-8.2)	
7. Nocturnal Sleep Final	0.285	1.389	-0.401	1.378	79.2	0.081	0.821	2.85%	
7a. Nocturnal Sleep modified	0.26	1.466	-0.399	1.209	56.4	0.118	0.761	2.95%	3.47%
8. Summary Scale Set up	0.06	2.319	-0.332	1.631	370.4	<0.001	0.936	10.79% (8.4-13.2)	
9. Summary scale Final	-0.077	1.173	-0.31	1.144	106.2	0.117	0.916	5.40%	6.62%
**Validation Sample**									
10. Physical	0.066	0.867	-0.337	1.098	62.9	0.77	0.905	6.03% (3.6-8.4)	7.23%
11. Cognitive	0.234	0.739	-0.358	0.994	22.8	0.74	0.842	3.15%	10.38%
12. Summary	0.16	1.329	-0.381	1.284	97.4	0.278	0.898	6.62% (4.2-9.0)	2.83%
13. Diurnal Sleep	0.041	1.158	-0.462	1.335	58.8	0.305	0.843	7.69% (5.3-10.1)	
14. Diurnal Sleep Modified	0.069	1.136	0.451	1.235	68.9	0.083	0.845	4.76%	4.09%
15. Nocturnal Sleep	0.235	1.817	-0.377	1.293	102.1	0.001	0.808	5.99% (3.6-8.4)	
16. Nocturnal Sleep modified	0.26	1.466	-0.399	1.209	56.4	0.118	0.761	3.15%	3.77%
***Ideal Values***	***0***	***<1.4***	***0***	***<1.4***		***>0.05***^a^	***>0.85***	***<5.0% (CI)***	

^a ^Bonferroni adjusted alpha level

PSI = person separation index; CI = confidence interval (only shown for values over 5%)

##### Physical scale

Rasch analysis of the 16 Physical items identified in the PCA indicated that all item thresholds were ordered, suggesting respondents could properly discriminate between response options. There was no DIF by age, gender, or duration of disease. The 16 item set displayed multidimensionality (Table 4, analysis 1), with 14.6% (CI 12.2-17.0%) of t-tests indicating significantly different person estimates derived from different subsets of items. An iterative process led to a scale reduction to 8 items. The resulting 8 item 'Physical' scale showed good fit to model expectations (Table 4, analysis 2) and just 4.13% of t-tests were significant, confirming a unidimensional scale.

##### Cognitive scale

All thresholds were ordered and DIF was absent. Overall, the original 8 items failed to meet model expectations (Table 4, analysis 3). Two items showed local dependency: 'mental effort really takes it out of me' and 'Having to concentrate for too long makes me feel weak'. This meant that these items were very similar, more-or-less measuring the same thing, and so one would be redundant, After removal of misfitting items, a four item scale satisfied model expectations (Table 4, analysis 4) with strict unidimensionality.

##### Relief by diurnal sleep or rest scale

The seven items from the diurnal sleep scale satisfied model expectations (Table 4, analysis 5). There was no local dependency, and the scale was strictly unidimensional. Two items showed DIF by gender: 'I need to rest in the day' and 'I try to rest or sleep beforehand, if I know I have to do something...'. These were biased in opposite directions with males more likely to report a higher score on the former, and females the latter. At the scale level, the DIF cancelled out.

##### Abnormal nocturnal sleep and sleepiness scale

All thresholds were ordered for the 8 item scale. One item, 'If I sleep in the day, I don't sleep well at night' displayed substantial misfit, and overall the scale failed to satisfy model expectations (Table 4, analysis 6). Removal of the misfitting item improved the overall fit of the scale, with no local dependency or DIF, and strict unidimensionality (Table 4, analysis 7).

##### Summary scale

All items from the subscales above were then included as potential items for a summary scale (a higher order factor). This resulted in significant misfit to model expectations and a clear multidimensional structure (Table 4, analysis 8). The items split into two groups, a physical-cognitive component, and a sleep-rest component. From the former, a 10 item summary scale was derived, satisfying all aspects of model expectation (Table 4, analysis 9). It was not possible to derive a summary scale for sleep, as the items consistently fractured into the two components of the diurnal and nocturnal sleep scales.

#### Validation Data

The data from the validation sample for each derived scale were then fitted to the Rasch model. The Physical, Cognitive, and Summary scales all demonstrated fit to model expectations, with ordered thresholds, no DIF for person factors, no local dependency and strict unidimensionality (Table 4, analyses 10-12). The two sleep scales required further modifications to adjust for misfit (nocturnal sleep) or multidimensionality (diurnal sleep)(Table 4, analyses 13 and 15). Satisfactory solutions were found for each scale (Table 4, analyses 14 and 16). There was no DIF by sample which further strengthened the validity of the fit across both the samples. The Physical, Cognitive, and Summary scales all achieved a level of reliability necessary for use in individuals.

#### Targeting

The final scales displayed acceptable person-item targeting with percentages of extreme scores of less than 5%, apart from the cognitive scale which had a small ceiling effect of 7.2% and the physical scale which had a ceiling effect of 7.7% (Table 4, final column).

#### Test-retest reliability

Retesting was performed between 2 and 4 weeks. The invariance of the scales over time were confirmed by the absence of DIF. Test-retest reliability was good, with correlation coefficients above 0.7 at 2-4 weeks for all scales (Table 5). In addition, there were no significant differences in the median scores at the two time points (Wilcoxon Signed Rank; p > 0.05).

**Table 5:** Test-retest comparisons.

Scale	Spearman rho*	Median Scores T1, T2 **
Summary	0.864	21, 20
Physical	0.852	17, 16
Cognitive	0.826	7, 6
Nocturnal sleep	0.837	8, 8
Diurnal sleep	0.796	11, 10

Spearman correlation coefficients and median scores for subscales and Summary scores over 2—4 week period.

* all p < 0.001

T1 = initial completion, T2 = retest at 2—4 weeks

** all differences, by Wilcoxon Signed Rank, non-significant (p > 0.05)

#### External construct validity

The correlations between the NFI-MS, and comparator measures, are shown in Table 6. Those correlations between directly comparable scales (*e.g*. cognitive to cognitive) were of the magnitude of 0.7.

**Table 6:** External construct validity.

Scale	Summary	Physical	Cognitive	Nocturnal sleep	Diurnal sleep
**Physical**	0.96				
**Cognitive**	0.85	0.71			
**Nocturnal sleep**	0.62	0.60	0.55		
**Diurnal sleep**	0.65	0.63	0.55	0.51	
**MFIS phys-8**	0.71	0.72	0.55	0.44	0.51
**MFIS cog-5**	0.58	0.48	0.69	0.46	0.37
**FSS-5**	0.71	0.71	0.57	0.43	0.54
**VAS**	0.67	0.67	0.52	0.50	0.46

Pearson correlation coefficients (Spearman for the VAS) between the Rasch derived person locations of the NFI-MS scales and the comparator scales.

p < 0.001 for all correlations.

MFIS = Modified Fatigue Impact Scale, FSS = Fatigue Severity Scale, VAS = visual analogue scale.

#### Raw score to interval scale conversion

Given fit to the Rasch model, Table 7 provides a simple conversion of the raw score for each scale, to its interval scale equivalent.

**Table 7:** Raw score to interval scale conversion table.

Raw Score	Summary Scale	Physical Scale	Diurnal Sleep Scale	Nocturnal Sleep Scale	Cognitive Scale
0	0.00	0.00	0.00	0.00	0.00
1	2.49	1.91	1.71	2.04	1.38
2	4.26	3.33	3.03	3.53	2.58
3	5.49	4.37	4.07	4.63	3.64
4	6.48	5.24	4.97	5.55	4.62
5	7.32	6.03	5.85	6.37	5.53
6	8.07	6.75	6.72	7.12	6.36
7	8.76	7.42	7.58	7.83	7.13
8	9.42	8.09	8.46	8.52	7.89
9	10.05	8.75	9.29	9.18	8.67
10	10.65	9.42	10.09	9.85	9.54
11	11.28	10.10	10.88	10.56	10.63
12	11.91	10.81	11.63	11.31	12.00
13	12.54	11.58	12.38	12.19	
14	13.20	12.38	13.16	13.38	
15	13.86	13.23	14.01	15.00	
16	14.55	14.14	14.99		
17	15.30	15.06	16.27		
18	16.05	15.99	18.00		
19	16.83	16.95			
20	17.64	17.93			
21	18.45	18.97			
22	19.29	20.22			
23	20.13	21.85			
24	21.03	24.00			
25	21.96				
26	22.98				
27	24.12				
28	25.53				
29	27.42				
30	30.00				

The conversions remain valid provided there are no missing data.

## Discussion

Fatigue is an important symptom in many chronic diseases, and can have a considerable impact upon lifestyle [37,38]. Despite this, the scales used in the measurement of MS fatigue in health outcome studies have been shown to fall short of current standards, partly indicative of the lack of a clear definition of the construct [11,12]. Concern about the quality of existing measures led to a new study which, using qualitative approaches, introduced a detailed definition of fatigue and a scale with an original item set reflecting that definition [6].

No *a priori *assumptions regarding the dimensionality of fatigue were imposed for the derivation of the item subsets from the qualitative work. However, a fundamental requirement for unidimensionality is an assumption of the Rasch model and this, together with the exploratory factor analysis, guided the eventual sub-scales of the NFI-MS. In practice, the resulting domains were in accordance with the conceptual dimensions found in the qualitative phase, including the notion that the sub-dimensions were part of a single, supraordinate theme of 'neurological fatigue'.

Fit of scale data to the Rasch model also allows for a transformation of the ordinal raw score to an interval scale latent estimate which, given appropriate distributions, can be used in parametric procedures. There is a straightforward ordinal to interval scale equivalence, courtesy of a special property of the Rasch model called specific objectivity,[39] and this has been provided in the nomogram of Table 7. This equivalence table is only valid provided there are no missing data in the raw scores of any new sample.

### Strengths and limitations

In this study the Neurological Fatigue Index (NFI-MS) has been developed to meet the most rigorous, modern psychometric qualities for measurement. A combination of factor analysis and Rasch analysis led to strictly unidimensional scales for physical and cognitive fatigue, as well as a short summary scale. These solutions were validated upon a set-aside or validation sample and thus can be considered robust with respect to their internal construct validity. The magnitude of correlations between the physical and cognitive components and appropriate comparator measures also give support to the external construct validity of the scales.

Understanding of the full processes involved in fatigue is still in its infancy [40]. The production of a definition of fatigue and its measurement therefore might be in itself a worthy goal, but it was envisaged from the outset that these would just be the necessary first steps to exploration of the pathophysiology of the symptom. Thus the focus of this development has been upon the impairment of function as opposed to the social impact of fatigue. Nevertheless, the multi-dimensional nature of fatigue in MS lends itself to an exploration of the role of fatigue in the more complex bio-psychosocial model as expressed though the International Classification of Functioning, Disability and Health (ICF)[41].

The use of factor analytical techniques on ordinal data, although widespread in psychology and health outcomes, nevertheless remains contentious [42,43]. We have attempted to overcome some of these limitations by using a polychoric correlation matrix as the basis of our exploratory analysis, and parallel analysis to determine significant eigenvalues, but have otherwise used the procedures available in SPSS which would be widely available. Our previous work on simulated multidimensional data has indicated that this is a reasonably robust approach for a simple exploration of factorial structures in polytomous data [33].

At the present time these data are only supportive of the validity of the scales within MS, and thus the instrument should be considered to be the NFI-MS. However, further work is underway to validate the item set in Stroke and MND. This may confirm the generic validity of the existing subscales, or it may be suggestive of alternative subscale structures. This is an empirical matter and, until further evidence is available, the label NFI-MS should be used.

### Future directions

Other future work could include the determination of imaging correlates and comparison of neurological fatigue experienced in MS and other diseases of the nervous system. This would be contingent upon the above validation studies in other conditions. Further validation of the sleep scales is also required, as these may form an important component of a bio-psychosocial model analysis. An understanding of the potential integral or adaptive roles of day and night sleep would be a high priority. Appropriate cross-cultural validation would allow the use of the NFI-MS as an outcome measure in internationally based clinical trials [28].

## Conclusion

The NFI-MS provides a brief and easy-to-use tool for the measurement of fatigue in MS. It was developed from the reported experience of fatigue by patients in accordance with the latest FDA guidelines for scale development. A short summary scale is available, but underlying components can also be measured. Fit to the Rasch measurement model was rigorously tested and was found to be reproducible. Such fit means that interval level scaling is available when change scores need to be calculated. The scales have specific validation for MS and can be used on patients of any age, sex, and duration.

### Implications for practice and research

It is suggested that the Summary scale would be useful in both a clinical setting and as an outcome measure in clinical trials and the different subscales would be suited to physiological and bio-psychosocial studies. Given fit to the Rasch model, the raw score is a sufficient statistic for identifying the (ordinal) level of fatigue in patients by simply adding up the raw score for the scale, which lends itself to convenient everyday use in a clinical setting. The ordinal-interval transformation could be used whenever parametric statistics are required. The NFI-MS is free for use in all Public Health and not-for-profit agencies, and can be obtained from the authors following a simple registration.

## Competing interests

The authors declare that they have no competing interests.

## Authors' contributions

RJM and CAY contributed to the design, implementation, and analysis of the study. JFP and AT contributed to the analysis of the study. All authors contributed to the writing of the manuscript, and all approved the final version.
